# Detection, Characterization, and Molecular Typing of Human *Mycoplasma* spp. from Major Hospitals in Cairo, Egypt

**DOI:** 10.1155/2014/549858

**Published:** 2014-11-23

**Authors:** Mirihan A. Metwally, Aymen S. Yassin, Tamer M. Essam, Hayam M. Hamouda, Magdy A. Amin

**Affiliations:** ^1^Department of Microbiology, National Organization for Drug Control and Research (NODCAR), Giza 12561, Egypt; ^2^Department of Microbiology and Immunology, Faculty of Pharmacy, Cairo University, Kasr Eleini Street, Cairo 11562, Egypt

## Abstract

Mycoplasmas are fastidious slow growing organisms lacking a cell wall and mostly isolated from the mucosal surfaces of the respiratory and genitourinary tracts. There is a dearth of information regarding clinical *Mycoplasma* spp. isolates among Egyptian patients. A total of 170 samples were collected from patients and apparently healthy personnel in local public hospitals in Cairo, Egypt. Isolation of *Mycoplasma* spp. was carried out using appropriate culture media and further identification was carried out by biochemical tests followed by serotyping using specific antisera. Confirmation was done by PCR for detection of different *Mycoplasma* spp. using genus-specific primers targeting 16S ribosomal RNA gene. Characterization of the antibiotic resistance and sensitivity pattern against different antimicrobials was carried out using disc diffusion test. The results indicated the presence of six *Mycoplasma* spp. in 22.94% of the samples. Mycoplasmas were detected more frequently in throat swabs than sputum. *Mycoplasma pneumoniae* was highly sensitive to macrolides and quinolones but less sensitive to aminoglycosides and tetracyclines. Molecular techniques were found to be of more rapid, highly sensitive, able to detect nonviable organisms, and cost effective. These results shed light on difficulties of *Mycoplasma* detection and the superiority of molecular techniques over culture.

## 1. Introduction

Mycoplasmas (mushroom form) are eubacteria included within the class Mollicutes (from latin mollis = “soft,” cutis = “skin”), which comprises the smallest and simplest self-replicating bacteria.* Mycoplasma* spp. possess distinctive features such as lack of a rigid cell wall envelope, sterol incorporation into their own plasma membrane, reduced cellular (0.3–0.8 *μ*m diameter), and genome sizes (0.58–2.20 Mbp). In addition, they are characterized by fastidious growth requirements, fried-egg or mulberry-shaped colonies on agar, and they are not affected by *β*-lactams [1–4]. Due to their reduced genome sizes, mycoplasmas exhibit restricted metabolic and physiological pathways for replication and survival [3, 4]. This explains why these bacteria display strict dependence to their hosts for acquisition of amino acids, nucleotides, lipids, and sterols as biosynthetic precursors [3–5].

Several species are pathogenic in humans, including* M. pneumoniae*, which is implicated in 20–40% of community acquired pneumonia,* M. genitalium,* and* M. hominis *which are involved in pelvic inflammatory diseases [6]. There is an evidence for* Mycoplasma* infections playing a role in Gulf war syndrome/illness.* M. fermentans *has been found in the blood of Gulf war veterans at a much higher rate than in the overall population [7, 8]. Mycoplasmas are thought to be responsible for a number of unexplained symptoms, especially chronic fatigue states.* M. salivarium, M. orale, M. buccale, M. faucium, *and* M. lipophilum* are part of the normal flora of the human oropharynx and are generally regarded as commensal organisms except in immunocompromised patients [9, 10].

Difficulties of* Mycoplasma* spp. diagnosis include but are not limited to the fact that they are usually overlooked as “viral infection”; the symptoms are neither specific nor diagnostic as well as difficulties in culturing the organism from clinical samples and its maintenance in vitro and the very long incubation period required (up to 21 days). In addition, the diagnostic laboratory tests are unreliable, as although serological tests of* Mycoplasma* are the mainstay of laboratory diagnosis, these tests lack the sensitivity and specificity due to the poor specific immune response of the host [11–13].

Due to the lack of information about* Mycoplasma* infections among Egyptian patients, this study was done in order to characterize the different species of* Mycoplasma* among patients admitted to public and university hospitals in Cairo, Egypt. Studying the distribution patterns of pathogens among patients admitted to local and university hospitals in Egypt and particularly the greater Cairo metropolis can be used as a measure for understanding the dissemination of pathogens, as a large number of the population, both local residents of Cairo and outside, relies on these hospitals due to socioeconomic factors. In addition, in this study, a comparison was established between traditional (cultural, biochemical, and serotyping methods) and the molecular methods for the detection of mycoplasmas.

## 2. Materials and Methods

### 2.1. Isolation, Identification, and Biochemical Testing

Specimens were collected from El-Omrania Sader, El-kasr El-Einy, Bolak, and Om El-Masrien hospitals (all are public and university hospitals in greater Cairo area) and El-Borg Laboratories (private clinical lab with several branches in Cairo). A total of 110 specimens were collected (35 throat swabs and 75 sputum samples) from apparently sick patients. All sick patients showed respiratory symptoms, like sore throat, hoarseness, coryza, sneezing or cough (upper respiratory tract) or shortness of breath, asthma, bronchitis, or pneumonia (lower respiratory tract). Throat swabs were collected from patients who showed sore throat symptoms and sputum samples were collected from patients who had asthma, bronchitis, or shortness of breath. A total of 30 specimens (10 throat swabs and 20 sputum samples) were collected from apparent healthy individuals randomly. Healthy individuals had no respiratory symptoms but were chosen based on being at high risk. They were chosen from hospitals' laboratories staff members, nurses, technicians, or workers in close contact with the patients. In addition, 30 Rota virus lyophilized vaccines samples were examined. All samples were collected during the period from January 2012 to January 2014.

All specimens and samples were examined for* Mycoplasma* spp. using pleuropneumonia-like organism broth and agar media (PPLO) (Difco, MI, USA). Culture and purification procedures were followed as previously described [10, 11, 14]. Purified isolates were maintained as agar strips (agar blocks) in sterile Bijou bottles and frozen at −20°C. Unopened plates were examined under stereo (dissecting) microscope (Leitz, Germany), where the surface of the medium was scanned to visualize the colonies. Digitonin sensitivity test was carried out to differentiate between* Mycoplasma* and* Acholeplasma *genera using filter paper discs impregnated with 0.2 mL of 1.5% (W/V) ethanol solution of digitonin and dried overnight.* Mycoplasma* spp. show digitonin sensitivity while* Acholeplasma* spp. are resistant [15]. Biochemical identification was used for further testing of* Mycoplasma *spp. Glucose fermentation, arginine deamination, urea hydrolysis tests, and serological detection using antiserum impregnated discs were performed as previously described [16–18].

### 2.2. Molecular Identification

Positive isolates were further confirmed by PCR amplification of the 16S rRNA gene using* Mycoplasma* specific primers: forward primer: 5′-GGGAGCAAACAGGATTAGATACCCT-3′ and reverse primer: 5′-TGCACCATCTGTCACTCTGTTAACCTC-3′, [19]. Positive* Mycoplasma pneumoniae* isolates were further typed by PCR amplification of the P1 cytadhesin gene using the forward primer: 5′-CCGCGAAGAGCAATGAAAAACTCC-3′ and reverse primer: 5′-TCGAGGCGGATCATTTGGGGAGGT-3′, [20]. For both PCR reactions, DNA extraction was done as previously described [21, 22]. The reaction was performed using 5 *μ*L of 10x PCR buffer, 4 *μ*L of 25 mM Mgcl_2_, 20 pmoles of each primer, 1 ng of DNA, 5 *μ*L of 200 *μ*M dNTP, and 1.25 units of Taq polymerase in final volume of 50 *μ*L using purified water. Amplification conditions were denaturation at 94°C for 10 min followed by 35 cycles of denaturation at 94°C for 1 min, primer annealing at 60°C (16S rRNA gene) or 58°C (P1 cytadhesin gene) for 1 min, and elongation at 72°C for 1 min; the cycling was followed by a final extension step at 72°C for 10 min. PCR reactions were purified using AxyPrep PCR clean-up kit (Axygen Biosciences, CA, USA); then sequencing reactions were performed by (MacroGen, MD, USA). Sequences were then submitted to NCBI GenBank using BankIt (http://www.ncbi.nlm.nih.gov/WebSub/?tool=genbank).

## 3. Results

### 3.1. Bacterial Isolates

Among the 140 total specimens collected from sick and healthy individuals,* Mycoplasma* spp. were found positive in 39 specimens (30%). Mycoplasmas were detected more frequently in throat swabs than sputum samples as a total of 14 isolates (11 isolates from apparently sick patients and 3 isolates from apparently healthy individuals) were detected in the 45 total throat swabs (31.1%), while 25 isolates (21 isolates from apparently sick patients and 4 isolates from apparently healthy individuals) were detected in the 95 total sputum samples (26.3%). All Rota virus vaccine samples (30 samples) were negative for* Mycoplasma* spp.

### 3.2. Culture Characters and Biochemical Tests

**Figure 1:**
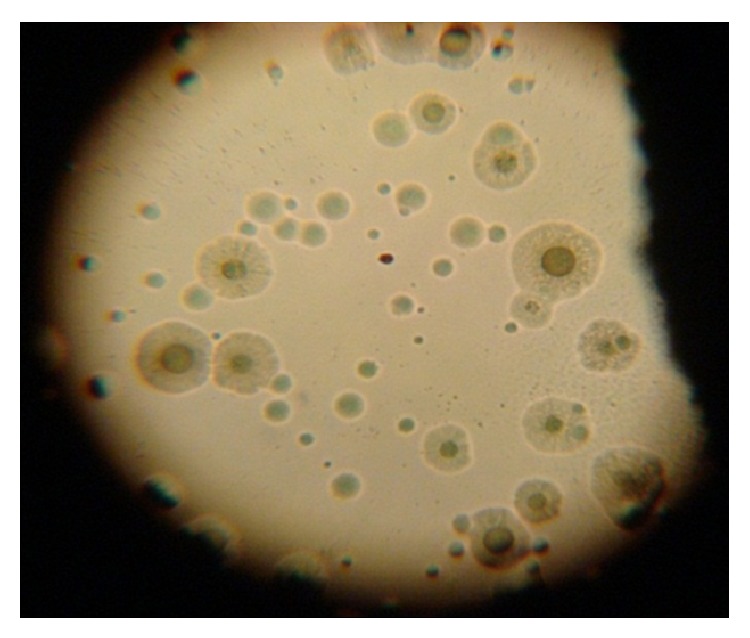
*Mycoplasma *colonies with fried-egg appearance.

Microscopic examination of the positive culture plates revealed spherical colonies, with fried-egg appearance—in which a dark central zone is usually surrounded by a lighter peripheral zone—or finely granular berry-like colonies that penetrate the agar surface (Figure 1); some* Mycoplasma* formed tiny pinpoint colonies. All of the 39 positive* Mycoplasma* spp. isolates showed inhibition zones > 3 mm in the digitonin sensitivity test. The isolates were then subjected to three biochemical tests (glucose fermentation, arginine deamination, and urea hydrolysis) where they were differentiated into three distinct groups. Further identification was carried out by testing growth inhibition using specific diagnostic antisera.  Table 1 summarizes the numbers and percentages of the different* Mycoplasma* spp. detected according to their classification by biochemical testing in each group.  Table 2 summarizes the different species of* Mycoplasma* according to their origin or source of isolation.

### 3.3. Molecular Detection

Direct detection of the* Mycoplasma* spp. was carried out by PCR of the 16S rRNA gene and using* Mycoplasma* spp. specific primers on the DNA purified from the isolates. All positive isolates showed an amplification band of 280 bp as expected.* M. pneumoniae* isolates were further confirmed by PCR amplification of the P1 cytadhesin gene where they showed an amplification band of 375 bp as expected. All PCR products were sequenced and the sequences were blasted against available sequences in the NCBI gene bank to confirm the identification of the different* Mycoplasma* spp. Four partial sequences were submitted to the NCBI gene bank where they received accession numbers: KJ561784 (*M. orale*), KJ561785 (*M. fermentans)*, KJ561786 (*M. hominis*), (16S rRNA gene), and KJ677969 (*M. pneumoniae*) (P1 cytadhesin gene partial sequence).

Disc diffusion test was carried on five* M. pneumoniae* isolates using different classes of antibiotics including quinolones (ciprofloxacin, norfloxacin, and enrofloxacin), macrolides (erythromycin), tetracyclines (tetracycline, doxycycline), aminoglycosides (streptomycin, gentamicin), and lincomycin. Zones of inhibition “sensitivity” were measured from the edge of the disk to the nearest area of growth. Our study revealed that* M. pneumoniae* was highly sensitive to macrolides (erythromycin), observed by the inhibition zone with the largest diameter for all five* M. pneumoniae* isolates, and followed by fluoroquinolones (ciprofloxacin, norfloxacin, and enrofloxacin) and tetracyclines (tetracycline and doxycycline). However, all five* M. pneumoniae* isolates were less sensitive (partially resistant) to aminoglycosides (streptomycin and gentamicin) and resistant to lincomycin (no inhibition zone).

## 4. Discussion

The study of* Mycoplasma* has become important in understanding chronic diseases. As both an extracellular and an intracellular pathogen, a better understanding of the virulence mechanisms of* Mycoplasma* spp. will provide fresh understanding of how to diagnose and combat this pathogen. The aim of this study was to screen respiratory samples for possible presence of* Mycoplasma* spp. and to conduct a comparative study between conventional and molecular methods for their detection among Egyptian patients. No doubt that rapid diagnosis of mycoplasmas leads to rapid choice and initiation of the most appropriate antimicrobial treatment and, consequently, rapid treatment and control of* Mycoplasma *infections avoiding its fatal complications.

Bacteriological examination was done for respiratory specimens (from both apparently sick and apparently healthy individuals) and vaccines samples collected during the period from January 2012 to January 2014. Different techniques were used for detection and identification of* Mycoplasma*. Culture technique included primary isolation, bacteriological examination, biochemical identification, and serotyping. Microscopic examination identified* Mycoplasma* according to colony appearance; then biochemical characterization was carried out followed by serological typing using growth inhibition test for the isolates in each biochemical group against specific antisera. In this study, 39 out of 170 samples (22.94%) were positive for* Mycoplasma,* where* Mycoplasma *spp. were detected more frequently in throat swabs (31.11%, in 14 out of 45 total swabs) than sputum (26.31%, in 25 out of the 95 total sputum specimens). All vaccine samples were negative for* Mycoplasma*. Rota virus vaccine is an example of a live attenuated vaccine so it has a high risk of* Mycoplasma* contamination.* Mycoplasma* contamination of cell culture (used for vaccine preparation) is a serious concern for biopharmaceutical industry. Contamination usually originates from components of cell culture medium such as serum or is introduced via individuals working in the laboratory or manufacturing facility. Consequently, any* Mycoplasma* contamination, if present, could indicate that insufficient care has been taken during vaccine manufacture or quality control. Sensitivity to digitonin was performed to differentiate the sterol requiring Mollicutes* (Mycoplasma, Ureaplasma, Entomoplasma, Spiroplasma, *and* Anaeroplasma) *from the non-sterol-requiring Mollicutes* (Mesoplasma, Acholeplasma, *and* Asteroleplasma)*. In agreement with previous studies, all positive isolates (39 isolates) were digitonin sensitive [23]. Six species of* Mycoplasma* were isolated, namely,* M. salivarium* 10/39 (25.64%),* M. orale* 10/39 (25.64%),* M. buccale* 7/39 (17.95%),* M. hominis* 6/39 (15.38%),* M. pneumoniae* 5/39 (12.82%), and* M. fermentans* 1/39 (2.56%).

Our results agree with previous work in which* M. salivarium* was found to be the most common* Mycoplasma* isolate obtained from human respiratory tract among other* Mycoplasma *spp. [10]. According to the same study, mycoplasmas isolated from humans were classified into widespread, common, and less common or rare;* M. salivarium* and* M. orale *are widespread;* M. hominis* is common while* M. buccale, M. pneumoniae,* and* M. fermentans* are less common. Many* Mycoplasma* spp. exist as commensals of the oropharynx,* M. salivarium* and* M. orale* being the most commonly found species [11]. In addition to the previous two* Mycoplasma* spp.,* M. buccale, M. facium*, and* M. lipophilium* are all part of the normal flora of the human oropharynx and are generally regarded as commensal organisms. In a previous study,* M. orale* has been detected in between 30 and 60% of throat swabs from adults, whereas* M. salivarium* has been detected in between 60% and 80% of swabs and* M. buccale*,* M. faucium,* and* M. lipophilum* were observed in <5% of cases [10]. The role of* M. orale *and* M. salivarium *in disease is limited to few reported infections, such as septic arthritis in immunocompromised hosts. In a previous survey carried out on bronchoalveolar lavage,* M. salivarium* was the most commonly* Mycoplasma* spp. isolate and was detected more frequently from HIV-positive cases (18%) than from HIV-negative (8%) [24].

In our study,* M. pneumoniae* accounted for 5/39 (12.82%) of the cases. The same frequency of isolation of* M. pneumoniae* coincided with a previous local study done in Egypt as well as another study done in Saudi Arabia [25, 26]. In Yemen,* M. pneumoniae* was recovered from pulmonary complications in 14.4% of patients [27]. In England and Wales one in seven children aged 5–14 years with respiratory signs tested positive for* M. pneumoniae* from October 2011 to January 2012 [28]. In all the previous studies, Bronchopneumonia and lobar pneumonia were the most frequent underlying clinical conditions among* M. pneumoniae* cases.* M. hominis* was found in 6/39 (15.38%) of positive* Mycoplasma* isolates, but it was not isolated from sputum or throat swabs of the apparently healthy group demonstrating that it could be a potential pathogen; the same observation was previously described in a study in which* M. hominis* was identified in 15/107 (14.01%) of positive* Mycoplasma* spp. isolates obtained from noncontrol clinically ill patients [19].* M. hominis* can colonize the human respiratory tract and has been found in respiratory secretions in up to 3% of healthy persons and in up to 6% of persons with chronic respiratory tract disease [29, 30].

In our study, characterization of the antibiotic resistance and sensitivity pattern against different antimicrobials using disc diffusion test was carried on* M. pneumoniae *isolates, using different classes of antibiotics; it was very challenging as plates were examined for inhibition zones existing around the disc to the distance where colonies start to appear, under the dissecting microscope as recommended [31]. Our study revealed that* M. pneumoniae* was highly sensitive to macrolides, followed by fluoroquinolones and tetracyclines. However, the isolates were less sensitive (partially resistant) to aminoglycosides and resistant to lincomycin. These results are in agreement with previous studies in which it was found that macrolides, tetracyclines, and fluoroquinolones eliminate mycoplasmas efficiently both in vivo and in vitro [32–34]. The antimicrobial agents of choice for treating lower respiratory tract* M. pneumoniae* infections are the macrolides in both adults and children [4].

Clinical presentation of* Mycoplasma* spp. is variable and diagnosis confirmation is a challenge to even the most experienced clinicians [13]. Bacterial culture was generally considered as the gold standard detection method of mycoplasmosis. Culture requires specialized media and is time consuming (up to 21 days). Due to the vast reduction in time in comparison with culture, PCR has been used increasingly for* M. pneumoniae *detection. Molecular methods have lessened the reliance on the problematic serological detection systems. Several gene targets have been used for amplification including the 16S ribosomal RNA gene, the elongation factor tuf, the P1 cytadhesin gene, and repetitive elements [35, 36].

In this study PCR was used for detection of* Mycoplasma* spp. using primers targeting a highly conserved region of 16S ribosomal RNA gene. The positive samples showed amplification products of 280 bp bands. In addition, PCR was used also for specific detection of* M. pneumoniae* P1 cytadhesin gene, where the positive samples showed amplification products of 375 bp bands on electrogram. This result was supported by other works in which PCR was investigated as a means of diagnosing* M. pneumoniae* infections [37]. The target DNA sequence was a 375 bp segment of the P1 virulence protein. This DNA segment was amplified from pure cultures of* M. pneumoniae* but not in other species of* Mycoplasma*,* Acholeplasma*, or* Ureaplasma.*

Development and application of molecular-based methods during the past two decades has significantly improved the ability to detect and identify mycoplasmas and ureaplasmas in clinical specimens, enabled expansion of knowledge about the diseases they may cause, and provided more rapid and accurate diagnosis. This study and other numerous studies indicated that an amplified DNA detection system was simple, rapid and combines maximum sensitivity with high specificity [38]. The future of diagnostic Mycoplasmology and epidemiological research rests with molecular-based technology.

## 5. Conclusion

A comparative study was done between molecular diagnosis and conventional diagnosis for detection of* Mycoplasma* spp. Six species of* Mycoplasma* existed among the population and samples studied. Characterization of the antibiotic resistance and sensitivity pattern against different antimicrobials revealed that macrolides are the drug of choice for treatment of* Mycoplasma* spp. infections. Molecular techniques were found to be more rapid, highly sensitive, detect nonviable organisms, and appeared to be cost effective. Culture methods and biochemical tests were found to be specific for detection of viable* Mycoplasma* spp.; however, it proved to be time consuming.

## Figures and Tables

**Table 1:** Classification of the isolated *Mycoplasma* species into three groups according to biochemical tests.

Groups	*Mycoplasma* species	Number of isolates	Percentages among total isolates
**Group I** Glucose positive Arginine negative Urea negative	*M. pneumoniae *	5	12.82%

**Group II** Glucose negative Arginine positive Urea negative	*M. hominis *	6	15.38%
	*M. orale *	10	25.64%
	*M. salivarium *	10	25.64%
	*M. buccale *	7	17.95%

**Group III** Glucose positive Arginine positive Urea negative	*M. fermentans *	1	2.56%

Total		**39**	**100**

**Table 2:** The number and percentages of each *Mycoplasma* species isolated from all specimens and samples.

	Positive isolates	Number and percentage of isolates											
		*M. pneumoniae *		*M. hominis *		*M. orale *		*M. salivarium *		*M. buccale *		*M. fermentans *	
		No.	%	No.	%	No.	%	No.	%	No.	%	No.	%
Patients													
Throat swab	11	2	18.18	2	18.18	3	27.27	2	18.18	1	9.09	1	9.09
Sputum	21	3	14.29	4	19.05	6	28.57	5	23.81	3	14.29	—	—
Healthy individuals													
Throat swab	3	—	—	—	—	—	—	1	33.33	2	66.67	—	—
Sputum	4	—	—	—	—	1	25.00	2	50.00	1	25.00	—	—

Total	**39**	**5**	**12.82**	**6**	**15.38**	**10**	**25.64**	**10**	**25.64**	**7**	**17.95**	**1**	**2.56**

## References

[1] Bové J. M. (1993). Molecular features of mollicutes. *Clinical Infectious Diseases*.

[2] Razin S., Yogev D., Naot Y. (1998). Molecular biology and pathogenicity of mycoplasmas. *Microbiology and Molecular Biology Reviews*.

[3] Chatterjee A., O'Keefe C., Johann-Liang R., Windle M. L., Lutwick L. I., Tolan R. W., Steele R. W. (2012). *Pediatric Mycoplasma Infections*.

[4] Giono-Cerezo S., Estrada-Gutiérrez G., Rivera-Tapia J. A., Yanez-Santos J. A., Diaz-Garcia F. J., Irusen E. M. (2012). Current status of the mollicute (Mycoplasma) lung disease: pathogenesis, diagnostics, treatment and prevention. *Lung Diseases—Selected State of the Art Reviews*.

[5] Baseman J. B., Tully J. G. (1997). Mycoplasmas: sophisticated, reemerging, and burdened by their notoriety. *Emerging Infectious Diseases*.

[6] Waites K. B., Xiao L., Paralanov V., Viscardi R. M., Glass J. I. (2012). Molecular methods for the detection of mycoplasma and ureaplasma infections in humans: a paper from the 2011 William Beaumont Hospital symposium on molecular pathology. *The Journal of Molecular Diagnostics*.

[7] Nicolson G. L., Nasralla M. Y., Nicolson N. L., Haier J. (2003). High prevalence of mycoplasma infections in symptomatic (chronic fatigue syndrome) family members of mycoplasma-positive Gulf War illness patients. *Journal of Chronic Fatigue Syndrome*.

[8] Nicolson G. L., Berns P., Gan R., Haier J. (2005). Chronic Mycoplasmal infections in Gulf war veterans' children and autism patients. *Medical Veritas*.

[9] McCormack W. M., Braun P., Lee Y. H., Klein J. O., Kass E. H. (1973). The genital mycoplasmas. *The New England Journal of Medicine*.

[10] Miles R. J., Nicholas R. A. J. (1998). *Methods in Molecular Biology*.

[11] Blanchard A., Bébéar C. M., Razin S. A., Herrmann R. (2002). Mycoplasmas of humans. *Molecular Biology and Pathogenicity of Mycoplasmas*.

[12] Talkington D. F., Shott S., Fallon M. T., Schwartz S. B., Thacker W. L. (2004). Analysis of eight commercial enzyme immunoassay tests for detection of antibodies to *Mycoplasma pneumoniae* in human serum. *Clinical and Diagnostic Laboratory Immunology*.

[13] Tharwat N., Naguib H., El-Nashar N., Mohie El-Dien S., Fouda A., Zedan M. (2010). Mycoplasma pneumoniae infection in children with acute exacerbation of bronchial asthma: clinical, laboratory and radiological evaluation. *Egyptian Journal of Bronchology*.

[14] Razin S., Tully J. G. (1983). *Textbook in Methods in Mycoplasmology*.

[15] Freundt E. A., Andrews B. E., Erno H., Kunze M., Black F. T. (1973). The sensitivity of *Mycoplasmatales* to sodium polyanetholsulfonate and digitonin. *Zentralblatt für Bakteriologie, Parasitenkunde, Infektionskrankheiten und Hygiene*.

[16] Erno H., Stipkovits L. (1973). Bovine mycoplasmas: cultural and biochemical studies. *Acta Veterinaria Scandinavica*.

[17] Krogsgaard-Jensen A. (1972). Mycoplasma: growth precipitation as a serodiagnostic method. *Applied microbiology*.

[18] Howard W. W., Ricardo F. C., Lioyd H. L. (1994). *Textbook of Mycoplasmosis in Animals*.

[19] El-Ebeedy D. A., Serry F. M., Kadry A. A., Ammar A. M. (2011). *Studies on the rule of Mycoplasma in causing some human infections [Ph.D. thesis]*.

[20] Ramirez J. A., Ahkee S., Tolentino A., Miller R. D., Summersgill J. T. (1996). Diagnosis of *Legionella pneumophila*, *Mycoplasma pneumoniae*, or *Chlamydia pneumoniae* lower respiratory infection using the polymerase chain reaction on a single throat swab specimen. *Diagnostic Microbiology and Infectious Disease*.

[21] Fan H. H., Kleven S. H., Jackwood M. W. (1995). Application of polymerase chain reaction with arbitrary primers to strain identification of *Mycoplasma gallisepticum*. *Avian Diseases*.

[22] Chávez González Y. R., Bascuñana C. R., Bölske G., Mattsson J. G., Molina C. F., Johansson K.-E. (1995). In vitro amplification of the 16S rRNA genes from *Mycoplasma bovis* and *Mycoplasma agalactiae* by PCR. *Veterinary Microbiology*.

[23] Poveda J. B., Miles R. J., Nicholas R. A. J. (1998). Biochemical characteristics in mycoplasma identification. *Methods in Molecular Biology*.

[24] Teel L. D., Finelli M. R., Johnson S. C. (1994). Isolation of *Mycoplasma* species from bronchoalveolar lavages of patients positive and negative for human immunodeficiency virus. *Journal of Clinical Microbiology*.

[25] El-Mofti M. F. M. (1993). *Association of common bacteria and viruses with lower respiratory tract infections in children in El- Shatby Hospital [Ph.D. thesis]*.

[26] Al-Rashed A. (1998). Role of *Mycoplasma pneumoniae* in acute respiratory-tract infections in Saudi paediatric patients. *Annals of Tropical Medicine and Parasitology*.

[27] Al-Moyed K. A., Al-Shamahy H. A. (2002). *Mycoplasma pneumoniae* infection in Yemen: incidence, presentation and antibiotic susceptibility. *Eastern Mediterranean Health Journal*.

[28] Chalker V., Stocki T., Litt D., Bermingham A., Watson J., Fleming D. M., Harrison T. G. (2012). Increased detection of *Mycoplasma pneumoniae* infection in children in England and Wales, October 2011 to January 2012. *Eurosurveillance*.

[29] Sackel S. G., Alpert S., Fiumara N. J., Donner A., Laughlin C. A., McCormack W. M. (1979). Orogenital contact and the isolation of *Neisseria gonorrhoeae, Mycoplasma hominis*, and *Ureaplasma urealyticum* from the pharynx. *Sexually Transmitted Diseases*.

[30] Pettersson B., Tully J. G., Bolske G., Johansson K. E. (2000). Updated phylogenetic description of the *Mycoplasma hominis* cluster based on 16S rDNA sequences. *International Journal of Systematic and Evolutionary Microbiology*.

[31] Lyon T. C., Nemes J. L. (1971). Antibiotic sensitivities of oral mycoplasma. *Journal of Dental Research*.

[32] Cassell G. H., Blanchard A., Duffy L., Crabb D., Waites K. B., Howard B. J., Keiser J. F., Weissfeld A. S., Smith T. F., Tilton R. C. (1994). Mycoplasmas. *Clinical and Pathogenic Microbiology*.

[33] Waites K. B., Bebear C. M., Robertson J. A., Talkington D. F., Kenny G. E. (2000). *Laboratory Diagnosis of Mycoplasma Infections. Cumitech 34*.

[34] Waltes K. B., Balish M. F., Atkinson T. P. (2008). New insights into the pathogenesis and detection of *Mycoplasma pneumoniae* infections. *Future Microbiology*.

[35] Nour M., Trabelsi A., Maatouk N., Hammami M. (2005). Amplification of P1 and 16S rRNA genes by nested PCR for detection of *Mycoplasma pneumoniae* in paediatric patients. *Pathologie Biologie*.

[36] Ross J. D. C., Jensen J. S. (2006). Mycoplasma genitalium as a sexually transmitted infection: implications for screening, testing, and treatment. *Sexually Transmitted Infections*.

[37] Buck G. E., O'Hara L. C., Summersgill J. T. (1992). Rapid, sensitive detection of *Mycoplasma pneumoniae* in simulated clinical specimens by DNA amplification. *Journal of Clinical Microbiology*.

[38] Amirmozafari N., Mirnejad R., Kazemi B., Sariri E., Bojari M. R., Darkahi F. D. (2009). Comparison of polymerase chain reaction and culture for detection of genital *Mycoplasma* in clinical samples from patients with genital infections. *Saudi Medical Journal*.

